# Commentary: Vitamin D status in relation to the clinical outcome of hospitalized COVID-19 patients

**DOI:** 10.3389/fmed.2022.977540

**Published:** 2022-08-12

**Authors:** Wael Hafez

**Affiliations:** ^1^NMC Royal Hospital, Khalifa City, Abu Dhabi, United Arab Emirates; ^2^The Medical Research Division, Internal Medicine Department, The National Research Center, Cairo, Egypt

**Keywords:** vitamin D, COVID-19, severity, mortality, polymorphism

## Introduction

Vitamin D is a crucial prohormone that mediates a variety of immunological responses. Numerous research findings revealed a strong association between vitamin D deficiency and a higher risk for communicable infections and poor outcomes that could be attributed to the role of vitamin D in regulating immunity (1).

While Controversies still persist concerning the plausible role of Vitamin D in COVID-19 disease severity and effect on the outcome; our study demonstrated a significant correlation between severe Vitamin D Deficiency and the risk of a poor disease outcome. We observed a significant link between severe vitamin D deficiency, ICU admission, and COVID-19-related in-hospital mortality (2).

In another study from the United Arab Emirates, serum 25(OH)D levels of 12 ng/mL were found to be highly linked with COVID-19 severity and mortality in a sample drawn from a similar population (3).

Another research on a different European population from 20 countries, reported that 25(OH)D concentrations and COVID-19 mortality were inversely correlated, and that vitamin D insufficiency was a poor prognostic factor for COVID-19 (4).

The reverse causality of the correlation between COVID-19 and the circulating 25(OH)D levels was also explored; Smolders et al. reported a decrease in the circulating 25(OH) D levels caused by the upregulation of the enzyme 25(OH) D1-alpha-hydroxylase as a result of the systemic inflammatory response associated with COVID-19 (5).

A retrospective research, investigates the association between pre-infection serum 25-hydroxyvitamin D [25(OH)D] levels and disease severity and death due to SARS-CoV-2, found that pre-infection vitamin D deficiency was associated with increased disease severity and mortality in hospitalized COVID-19 patients (6).

A meta-analysis and GRADE review of cohort studies and RCTs, on the other hand, suggested that vitamin D deficiency or insufficiency was not substantially linked to susceptibility to COVID-19 infection or death. The authors further argued that vitamin D supplementation did not improve clinical outcomes in COVID-19 patient (7). Given that supplementation studies are heterogenous in design, controversial results are again anticipated.

Genomics-guided tracing research found that vitamin D is involved in regulating gene expression and has the capability to minimize SARS-CoV-2 infection by binding to the vitamin D response element (8).

## Discussion

In a commentary on our study, Speeckaert M and Delanghe J highlighted the potentially essential role of vitamin D binding protein (DBP) and its polymorphism on the link between low vitamin D levels and poor COVID-19 outcome (9).

DBP is the main transporter and reservoir for the major vitamin D metabolites which are largely protein bound. There is a significant DBP polymorphism [DBP1S (slow), DBP1F (fast), and DBP2], as well as more than 120 uncommon variants (10).

The DBP can be defined by the genetic polymorphisms rs7041 and rs4588 as the C-allele of rs2282679 is related with lower 25(OH)D and DBP levels (11).

The observed link in our study could be partially explained by the effect of DBP polymorphism, as carriers of a DBP polymorphism corresponding with lower vitamin D and DBP concentrations might have a higher risk for poor prognosis (9). Furthermore; the polymorphism DBP rs2282679 might account for the majority of the intriguing link as another study suggested (12).

Given the debate over the impact of vitamin D status, other researchers found no significant link between vitamin D status and COVID-19 outcome (7). Based on these findings and trying to fit possible explanation, it is very plausible that some genetic factors/polymorphisms may influence vitamin D levels and/or function.

These polymorphisms are not just connected to DBP polymorphism, but also include polymorphisms in intermediate metabolites in the vitamin D pathway, vitamin D receptors, and enzymes impacting vitamin D catabolism (13).

It could be that while Vitamin D status is sufficient as per our definitions and reference ranges, but due to a polymorphism in vitamin D Receptors- VDR polymorphisms- the function of vitamin D is disrupted, abolished, or minimized, resulting in a status similar to functional vitamin D deficiency, or another polymorphism in the catabolism of vitamin D pathway leading to increased clearance of vitamin D, thereby affecting vitamin d function.

Several polymorphisms in genes associated with vitamin D metabolism have been identified as possible risk factors for severe COVID-19 outcomes (14, 15).

Analyses of genotype data in connection to vitamin D levels revealed the role of vitamin D homeostasis and its metabolic pathway in determining susceptibility to severe COVID-19 disease. The effect of vitamin D in host immunity against SARS-CoV-2 and other viral infections may explain these genotypic disparities in COVID-19 disease outcome (16, 17).

Al Anouti et al. investigated the genetic contribution of specific haplotypes for VDR, DHCR7/NADSYN1, and GC genes in to COVID-19 disease severity among the UAE population in a study that focused on the associations between genetic variants in the Vitamin D metabolism pathway and severity of COVID-19. The AA genotype in SNP rs59241277, the CC genotype in SNP rs113574864, the GG genotype in SNP rs182901986, the TT genotype in SNP rs60349934, and the GG genotype in SNP rs113876500 in gene GC, for example, were all linked to the critical COVID-19 condition (13).

Vitamin D metabolism is also mediated by several cytochrome P450 enzymes. CYP2R1 is one of the enzymes involved in vitamin D hydroxylation (18).

Several studies have been conducted to examine the relationship between CYP2R1 genetic variants and vitamin D status in different populations, and these investigations concluded that a strong correlation existed between specific polymorphisms on SNPs (rs10766197 and rs10741657) and the risk of vitamin D deficiency (19, 20).

On the other hand, Apaydin et al. found that 25 (OH)D levels were unrelated to COVID-19 severity and mortality, while VDR gene polymorphisms were significantly correlated with COVID-19 severity and patient survival (21).

The majority of the people in our survey were South Asians, not Arabs. One study from Kuwait found that CYP2R1 SNPs (rs10500804 and rs12794714) were substantially linked with serum 25(OH)D levels in the Arabian group but not in the South Asians (22). The same results were discovered in another study derived from a similar population to our study (13).

This could be explained by several complicated interactive effects of distinct polymorphisms in the vitamin D metabolic pathway, which significantly impact both its level and function.

This result suggests that not only DBP variations, but also all vitamin D Metabolic pathway associated genes, might have a role in COVID-19 disease prognosis and transmission. One plausible explanation- at least partially -for the debates around vitamin D's link to COVID-19 outcome could be the effect of such various polymorphisms in each study cohort on altering this effect among different populations. Even supplementation responses could be modulated by the genetic variations with DBP and other Vitamin D metabolism genes. The effect of Common polymorphisms of DBP on vitamin D supplementation was studied by Al-Daghri et al., who concluded that 25[OH]D concentrations were significantly higher among people with the major homozygous rs7041 genotype while 25[OH]D was higher in participants carrying homozygous major genotypes in rs4588 and rs7041 compared to other genotypes after supplementation (23).

Furthermore, the optimal level of vitamin D may range from one community to others dependent on the distribution of such polymorphisms in different populations.

These probable causes should be acknowledged for future research, and vitamin D effect could be appropriately examined in the context of the distribution of vitamin D metabolism related genes variation in different populations.

## Conclusion

While DBP polymorphisms may be involved in the link between vitamin D status and COVID-19 outcome, many other polymorphisms in Vitamin D metabolic pathway genes might also be involved, and future research should acknowledge investigating vitamin D status in the context of such polymorphism distribution in each study population, and proper vitamin D levels should be estimated taking these polymorphisms into consideration.

## Author contributions

The author confirms being the sole contributor of this work and has approved it for publication.

## Conflict of interest

The author declares that the research was conducted in the absence of any commercial or financial relationships that could be construed as a potential conflict of interest.

## Publisher's note

All claims expressed in this article are solely those of the authors and do not necessarily represent those of their affiliated organizations, or those of the publisher, the editors and the reviewers. Any product that may be evaluated in this article, or claim that may be made by its manufacturer, is not guaranteed or endorsed by the publisher.
